# Supplementary material for: Deletion of wfs1 Impairs Oligodendrocyte Precursor Cells Dorsal Distribution and Myelination Through the wfs1-hmgcs1 Axis in Zebrafish
Source: Biology (Basel). 2026 Aug 16;15(16):1402. doi: 10.3390/biology15161402 (PMC13510151; doi:10.3390/biology15161402)
Supplement: Supplementary file 1 [file biology-15-01402-s001.zip › File S1-Transcriptome sequencing data and differentially expressed genes (DEGs) in wfs1-- zebrafish at 5 dpf/2.KEGG/wfs1KOvsWT/UP/src/dre01240.html]

dre01240


- dre:572070

- Up regulated genes

110438097(4.95899171594655)

- dre:571967

- Up regulated genes

571967(1.93014769457854)

- dre:406789

- Up regulated genes

406789(1.01145231875249)

- dre:100330410

- Up regulated genes

100330410(1.88154010673068)
- dre:574009

- Up regulated genes

574009(1.57400981620651)

- dre:557552

- Up regulated genes

557552(1.27169280646928)

- dre:557552

- Up regulated genes

557552(1.27169280646928)

- dre:557552

- Up regulated genes

557552(1.27169280646928)

- dre:445287

- Up regulated genes

445287(1.98523130799607)
- dre:394142

- Up regulated genes

394142(1.09942837424348)

- dre:334195

- Up regulated genes

334195\_1(2.11046533957304)

- dre:445326

- Up regulated genes

445326\_1(2.09813524726464)

- dre:403070

- Up regulated genes

403070\_1(2.42915861580394)

- dre:334431

- Up regulated genes

334431\_1(1.27207238534102)

- dre:450084

- Up regulated genes

450084\_1(4.00880053586154)

- dre:368239

- Up regulated genes

368239(1.14034759711527)

- dre:393580

- Up regulated genes

393580(1.34154931476468)

- dre:445083

- Up regulated genes

445083(5.52673269190901)

Close
